# HPV Serology and Circulating Viral DNA for Detection, Genotyping, and Measurement of Disease Burden in Oropharyngeal Cancer

**DOI:** 10.1002/ijc.70619

**Published:** 2026-06-30

**Authors:** Lucas Penny, Eric Yang Stutheit‐Zhao, Birgitta E. Michels, Fabian Rosing, Zhen Zhao, Yangqiao Zheng, Jinfeng Zou, Shao Hui Huang, Johnny Carlton, Andrew McPartlin, John R. de Almeida, David Goldstein, Andrew Hope, Ali Hosni, John Kim, Fei‐Fei Liu, C. Jillian Tsai, John N. Waldron, Anna Spreafico, Enrique Sanz‐Garcia, Lillian L. Siu, Tim Waterboer, Geoffrey Liu, Scott V. Bratman

**Affiliations:** ^1^ Department of Medical Biophysics Temerty Faculty of Medicine, University of Toronto Toronto Ontario Canada; ^2^ Princess Margaret Cancer Centre, University Health Network Toronto Ontario Canada; ^3^ Department of Radiation Oncology, Temerty Faculty of Medicine University of Toronto Toronto Ontario Canada; ^4^ Infections and Cancer Epidemiology Division German Cancer Research Center (DKFZ) Heidelberg Germany; ^5^ Department of Otolaryngology‐Head & Neck Surgery/Surgical Oncology Princess Margaret Cancer Centre Toronto Ontario Canada; ^6^ Division of Medical Oncology & Haematology, Department of Medicine Princess Margaret Cancer Centre, University Health Network, University of Toronto Toronto Ontario Canada

**Keywords:** circulating tumor DNA (ctDNA), HPV serology, oropharyngeal cancer, risk‐stratification

## Abstract

The incidence of human papillomavirus‐positive (HPV+) oropharyngeal cancer (OPC) has increased rapidly, and HPV early antigen serology has been proposed as a scalable and cost‐effective early detection test. HPV seropositivity can precede clinical presentation of OPC by several years, so additional surveillance procedures may be necessary to optimize early cancer detection. The potential for HPV circulating tumor DNA (ctDNA) to confirm a diagnosis of OPC in seropositive individuals is poorly understood. Here, we assess the relationship between HPV serology and HPV ctDNA with disease burden in a large cohort of HPV+ OPC. We analyzed baseline blood samples from 262 patients using a multiplex serology assay and an HPV‐targeted deep sequencing assay (HPV‐seq), and HPV‐seq results were validated by an orthogonal droplet digital PCR assay. Both assays identified HPV16 as the most prevalent genotype (84%), with serology results indicating a total of 4 HPV genotypes and HPV ctDNA results indicating a total of 6 HPV genotypes. HPV ctDNA results demonstrated higher sensitivity and lower cross‐reactivity between HPV types compared with HPV serology results. Furthermore, HPV ctDNA but not HPV16 E6 antibody levels were positively associated with disease burden as determined by N‐category, overall stage, and by tumor volume (ctDNA vs. volume, *r* = 0.48 *p* = 6.4e‐12; E6 vs. volume, *r* = −0.079 *p* = 0.26). Overall, this is the largest cohort to compare HPV serology and HPV ctDNA results in OPC, and these findings highlight the potential for ctDNA to augment future strategies for blood‐based early detection of HPV+ OPC.

## Abstract (highlights)

What's new?

Both serology and ctDNA have been proposed as blood‐based biomarkers for early detection of HPV+ oropharyngeal cancer. In this large cohort study, a multiplexed sequenced‐based ctDNA assay outperformed serology for sensitive detection of disease burden. The emergence of ctDNA after a positive serology result potentially reflected low‐burden disease that may be treated earlier. HPV16 E6 antibody levels were lower in some patients with more advanced locoregional disease in whom HPV ctDNA levels were readily detectable. The study also suggests that HPV genotypes other than HPV16 may be distinguished by interrogating ctDNA, which could have further prognostic implications in oropharyngeal cancer.

## Graphical abstract

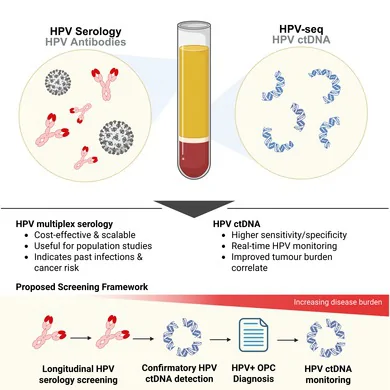

**Abbreviations**
AJCC: American Joint Committee on Cancer
ANOVA: analysis of variance
ctDNA: circulating tumor DNA
ddPCR: droplet digital PCR
DNA: deoxyribonucleic acid
ELISA: enzyme‐linked immunosorbent assay
GTV: gross tumor volume
HPV: human papillomavirus
HPV‐seq: HPV‐targeted deep sequencing
MFI: median fluorescence intensity
NLR: neutrophil‐to‐lymphocyte ratio
OPC: oropharyngeal cancer
TNM: tumor, node, metastasis
TNM‐8: 8th edition of the tumor, node, metastasis classification system
UHN: University Health Network
UICC: Union for International Cancer Control

## 1 Introduction

**FIGURE 1:**
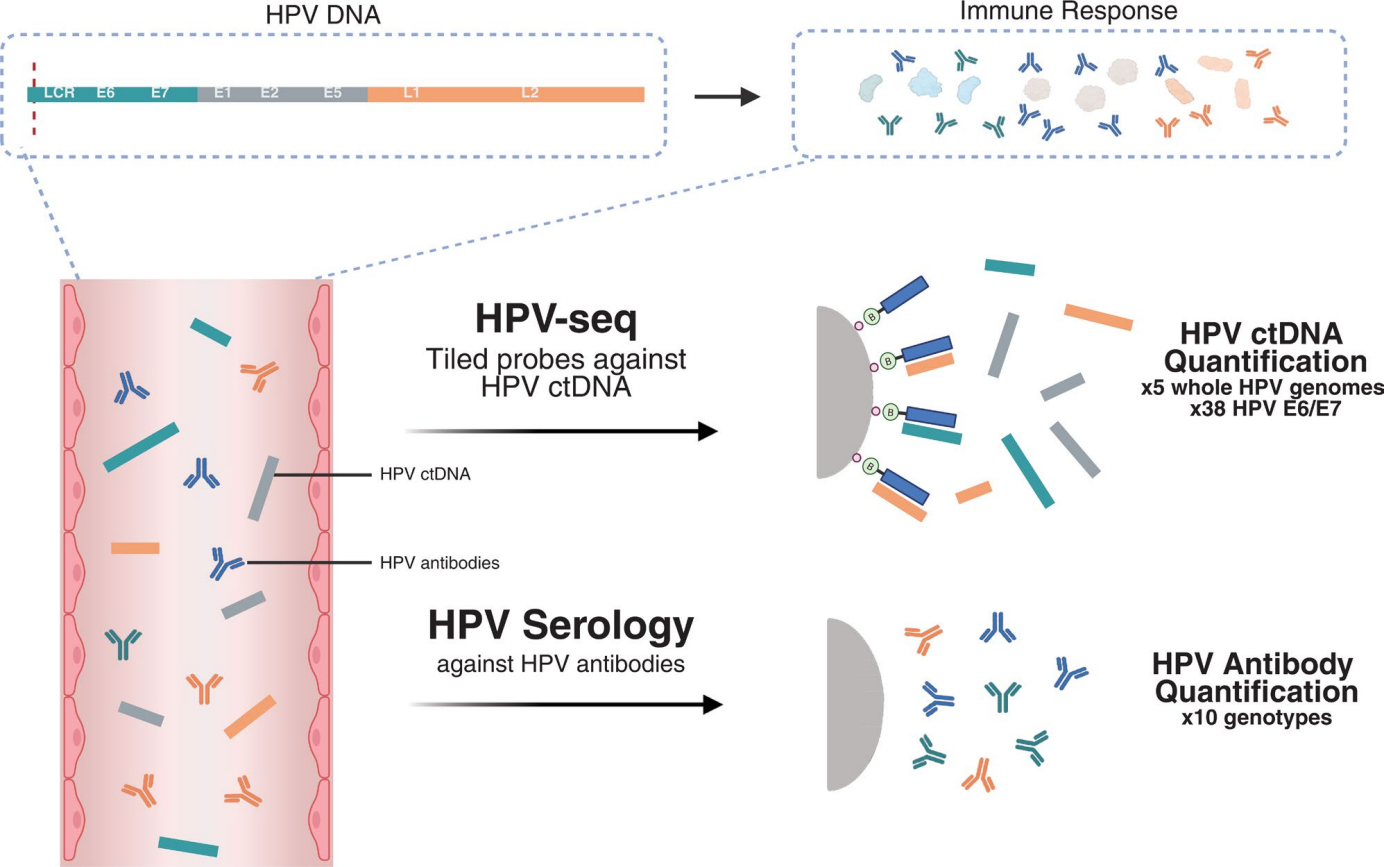
HPV‐seq & serology analysis overview. HPV ctDNA and antibodies in the plasma are captured and quantified. HPV‐seq captured 5 whole HPV genomes (HPV 16, 18, 31, 33, 35) and 38 genotypes from the E6 and E7 genes. A Multiplex Serology assay captured various HPV antibodies for 10 HPV genotypes. For each assay, the dominant genotype was selected and quantified for each patient (Methods).

The incidence of oropharyngeal cancer (OPC) has increased rapidly over recent decades, largely due to the causative effects of human papillomavirus (HPV) [1]. HPV infects epithelium in the cervix, oropharynx, oral cavity, and anogenital tract [2]. The proportion of HPV‐infected individuals who go on to develop OPC is low and can vary largely by HPV type as well as a number of environmental exposures [3]. Several HPV genotypes are considered high‐risk due to their oncogenic activity; HPV16 is most commonly associated with OPC; however, HPV35, HPV33, and HPV18 are also observed in a subset of cases [4]. Antibodies against multiple viral antigens are generated in response to acute and chronic infection and persist in the bloodstream of most individuals who eventually develop HPV‐driven malignancy [5]. As tumors progress, rapid cell turnover results in release of cellular remnants including tumor‐derived DNA into the bloodstream [6] (Figure 1).

**FIGURE 2:**
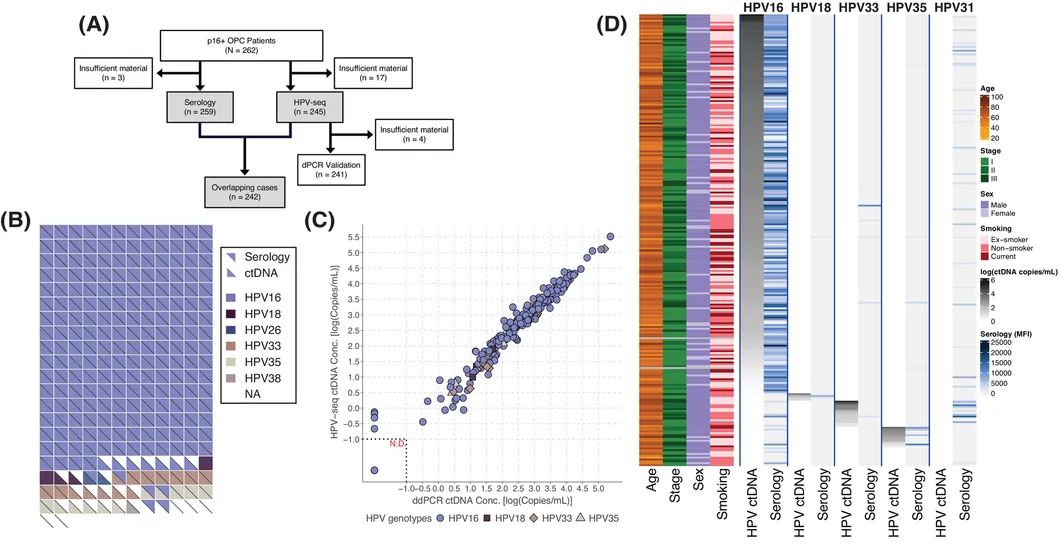
HPV detectability and genotyping using serology and HPV‐seq ctDNA. (A) CONSORT diagram. Patients were excluded from this study if both serology and HPV‐seq were not completed. (B) Each patient is represented by one box in the graph; the upper and lower triangle for each box reflects the dominant genotype detected by serology and HPV‐seq, respectively. If no genotype was detected in the assay, the plot was left blank. (C) HPV‐seq and ddPCR quantification are highly concordant (*R* ^2^ = 0.973). Plotted using log‐transformed quantities (y = log(x + 0.01)) to highlight the differences in limit of detection between HPV‐seq and ddPCR. Dotted line represents samples that had no HPV detected (N.D., not detected). (D) Heatmap representation of the dominant HPV ctDNA and serology signal for patients with HPV+ OPC. Clinical characteristics are represented to the left of the quantifications, and the 5 most common genotypes detected are represented on the right.

**FIGURE 3:**
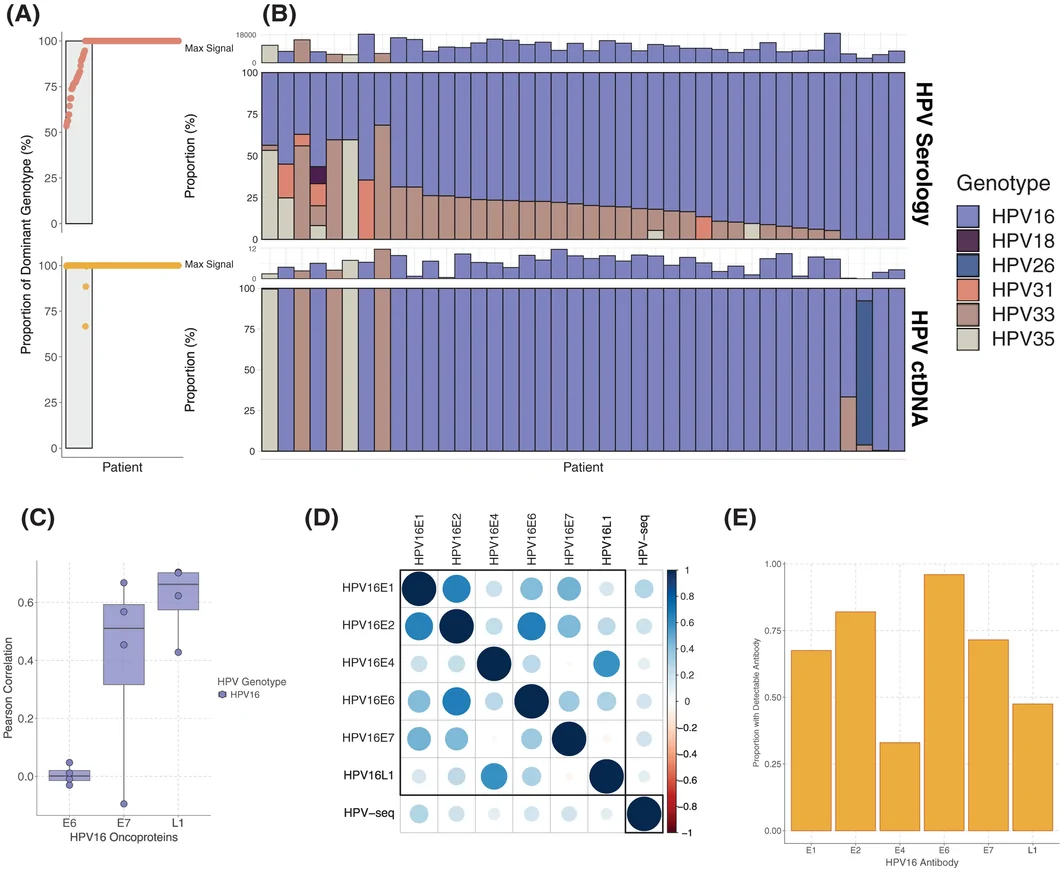
HPV serology antibodies show lower sensitivity and cross‐reactivity compared to HPV‐seq ctDNA detection. (A) Per‐patient proportion of total signal derived from the dominant HPV genotype across serology (top) and HPV‐seq (bottom). Higher values indicate greater single‐genotype dominance. Gray boxes indicate patients shown in panel (B). (B) Genotype distribution in patients with multiple HPV genotypes detected (patients highlighted in panel A). Top marginal plots show signal magnitude for each patient (serology: MFI; HPV‐seq: Log(counts/mL + 0.01)). (C) HPV16 antibody cross‐reactivity with other genotypes (HPV18, 31, 33, and 35). Each dot represents a HPV genotype. Box plots show median and interquartile range of antibody correlations for each genotype. (D) Pairwise correlations between HPV genotypes detected by serology and HPV‐seq. Circle size and color intensity indicate correlation strength (Pearson correlation coefficient). (E) Among patients with detectable HPV16 ctDNA by HPV‐seq, the proportion of seropositive patients for each HPV16 antibody target is shown.

Upon diagnosis of HPV+ OPC, prognosis and treatment options depend on the size and anatomical distribution of disease [7]. Improved tools are needed to identify individuals with early‐stage, low‐burden disease when outcomes are favorable and treatment more tolerable. Antibodies against early viral antigens have been proposed for the sensitive detection of early‐stage OPC. For example, the presence of HPV16 E6 antibodies in the bloodstream of North American men had a 10‐year risk of 17%–27% for developing OPC with a negative predictive value of > 99.9% [8]. Most HPV+ OPC patients with detectable HPV E6 antibodies will also have other detectable early antigen antibodies, such as E1, E2, and E7 (Figures 2 and 3).

One challenge with HPV antibody testing is that the 10‐year positive predictive value for detecting HPV+ OPC remains low at 1% [9] highlighting a need for diagnostic or surveillance procedures to confirm cancer status. Advances in next‐generation sequencing have propelled the use of plasma circulating tumor DNA (ctDNA) to improve cancer diagnosis and management. HPV ctDNA is also detectable in the blood and has shown promise in applications such as detection and risk stratification in cervical cancer [10, 11]. However, the potential for HPV ctDNA to confirm a diagnosis of OPC in seropositive individuals is poorly understood. Moreover, the concordance of baseline HPV antibody and HPV ctDNA and their respective associations with cancer burden in OPC patients is not known.

In this study, we investigated HPV serology and ctDNA results in a large cohort of OPC patients. We compared the performance of the two analytes for detecting the causative HPV genotype and measuring disease burden. Our results illustrate the complementarity of serology and ctDNA in newly diagnosed OPC.

## 2 Methods

### 2.1 Patient Recruitment

Pre‐treatment peripheral blood plasma was taken from 262 patients older than 18 years of age with non‐metastatic p16+ OPC treated with definitive (chemo)radiotherapy at University Health Network (UHN). Immunohistochemistry for p16 was used as part of routine clinical care to determine HPV association as previously described [12]. Patient samples were obtained between 2014 and 2019. TNM‐8 clinical stage was classified using the American Joint Committee on Cancer/Union for International Cancer Control (AJCC/UICC) 8th edition staging system.

### 2.2 Sample Collection and ctDNA Processing

For ctDNA processing, peripheral blood samples were collected from all participants in EDTA tubes and processed within 2 h of collection. Plasma was separated by centrifugation at 2500 × g for 10 min at 4°C, followed by a second centrifugation at 16,100 × g for 10 min at 4°C to remove residual cells and cellular debris. Plasma aliquots were immediately stored at −80°C until analysis. All samples were processed according to a standardized laboratory standard operating procedure to ensure consistency across the study cohort.

### 2.3 HPV ctDNA Sequencing Processing

Plasma cell‐free DNA was extracted and assessed for quality as previously described [13]. Cell‐free DNA was extracted from 2 to 5 mL of plasma per patient sample, and up to 10 ng of purified cell‐free DNA was used as input for HPV‐seq library preparation. HPV ctDNA was captured utilizing a custom HPV‐targeted hybrid capture panel for five whole viral genomes and 33 other HPV genotypes targeting the E6 and E7 genes (Lockdown probes, Integrated DNA Technologies Inc., Iowa, USA). Paired‐end sequencing for the target HPV‐seq panel was performed on the Illumina NovaSeq X Plus platform for 245 patient plasma samples. Raw sequencing data were demultiplexed, trimmed of adapters, aligned to Hg19 using BWA (v0.7.5), and then processed to remove duplicates using ConsensusCruncher [14]. Only uniquely mapped, non‐duplicate reads with mapping quality ≥ 30 were retained for downstream analysis. The sequencing coverage and quality statistics for each sample are summarized in Table S3.

### 2.4 HPV ctDNA Quantification

HPV genotype‐specific quantification was performed using HPV‐seq and ddPCR as previously described [13]. Quantification of HPV ctDNA fragments was performed using GATK (v3.8) “DepthOfCoverage” on properly paired reads of all.unique.dcs bam files. For HPV‐seq, a dominant genotype was determined for each patient by selecting the HPV genotype that had the majority of reads aligned to its respective genome. All but two samples had at least 99.4% of their reads aligned to one genome. For the other two patients, only 6 (HPV16: 4 reads; HPV31: 2 reads) and 52 HPV (HPV16: 6 reads; HPV26: 46 reads) reads were aligned to each patient, accounting for the low percentage.

A factor of 0.6 was applied to account for inflation of HPV‐mapped reads relative to human‐mapped reads because of incorporation of dual‐stranded HPV hybrid capture baits, as previously described [10]. From the 242 patients included in our study, 238 were validated by ddPCR. In cases where samples had undetectable HPV16 ctDNA levels, HPV‐seq was used to confirm the genotype and re‐run on ddPCR. Samples were considered positive if any HPV genotype was detected by the bioinformatics pipeline (i.e., > 0 reads mapping to the viral genome). Samples with no detectable reads mapping to the HPV genome were classified as negative.

HPV‐negative SW48 cell line DNA was included as a negative control in each sequencing batch to monitor for contamination and establish the limit of blank. All SW48 controls consistently showed no detectable HPV DNA across all batches. Additionally, to validate assay specificity, plasma samples from 50 healthy individuals with no history of cancer were analyzed using HPV‐seq.

### 2.5 HPV Status Determination

HPV status was determined using a standardized, validated institutional algorithm implemented by our institution [12]. p16 immunohistochemistry was performed on all oropharyngeal tumor specimens, with positivity defined as strong, diffuse cytoplasmic and nuclear staining in > 70% of tumor cells. For equivocal cases showing 50%–70% staining, high‐risk HPV DNA status was confirmed by in situ hybridization targeting HPV16 and HPV18, or in select cases by PCR. This stringent approach has resulted in an institutional p16/HPV discordance rate of 1.5% [15], among the lowest reported in the literature. All patients included in this study had p16‐positive oropharyngeal squamous cell carcinoma confirmed by this protocol.

### 2.6 Serology

For the multiplex serological assay, plasma samples were sent to the German Cancer Research Center (DKFZ, Heidelberg, Germany) and performed as previously described [16]. Briefly, the assay required 30 μL of serum per patient sample for detection of antibodies against HPV proteins. This assay is based on an enzyme‐linked immunosorbent assay (ELISA) in combination with fluorescent bead‐based suspension array technology to measure the levels of serum HPV antibodies (L1, E1, E2, E4, E6, E7) from 10 HPV genotypes and was quantified as median fluorescence intensity (MFI) units. Of note, not all genotypes had all six antibodies tested. Seropositivity, seronegativity, and serology quantification were determined adapted from a pre‐computed algorithm [17], which can be found in Table S4. For each patient, a dominant HPV genotype was determined by taking the highest genotype‐specific value from the algorithm.

### 2.7 Volumetric Analysis

Gross tumor volume was determined for each patient by a radiation oncologist specialized in the treatment of head and neck cancers. The gross tumor volume of the primary tumor and nodal tumors was determined by taking the volume (in cubic centimeters) of the primary tumor or the sum of all nodes larger than 1 cm^3^, respectively.

### 2.8 Statistical Analysis

Statistical analysis was performed in R (v4.1.1). Data manipulation operations were performed using data.table (v1.14.8), readxl (v1.4.2), tidyverse (v2.0.0), and dplyr (v1.1.2).

**FIGURE 4:**
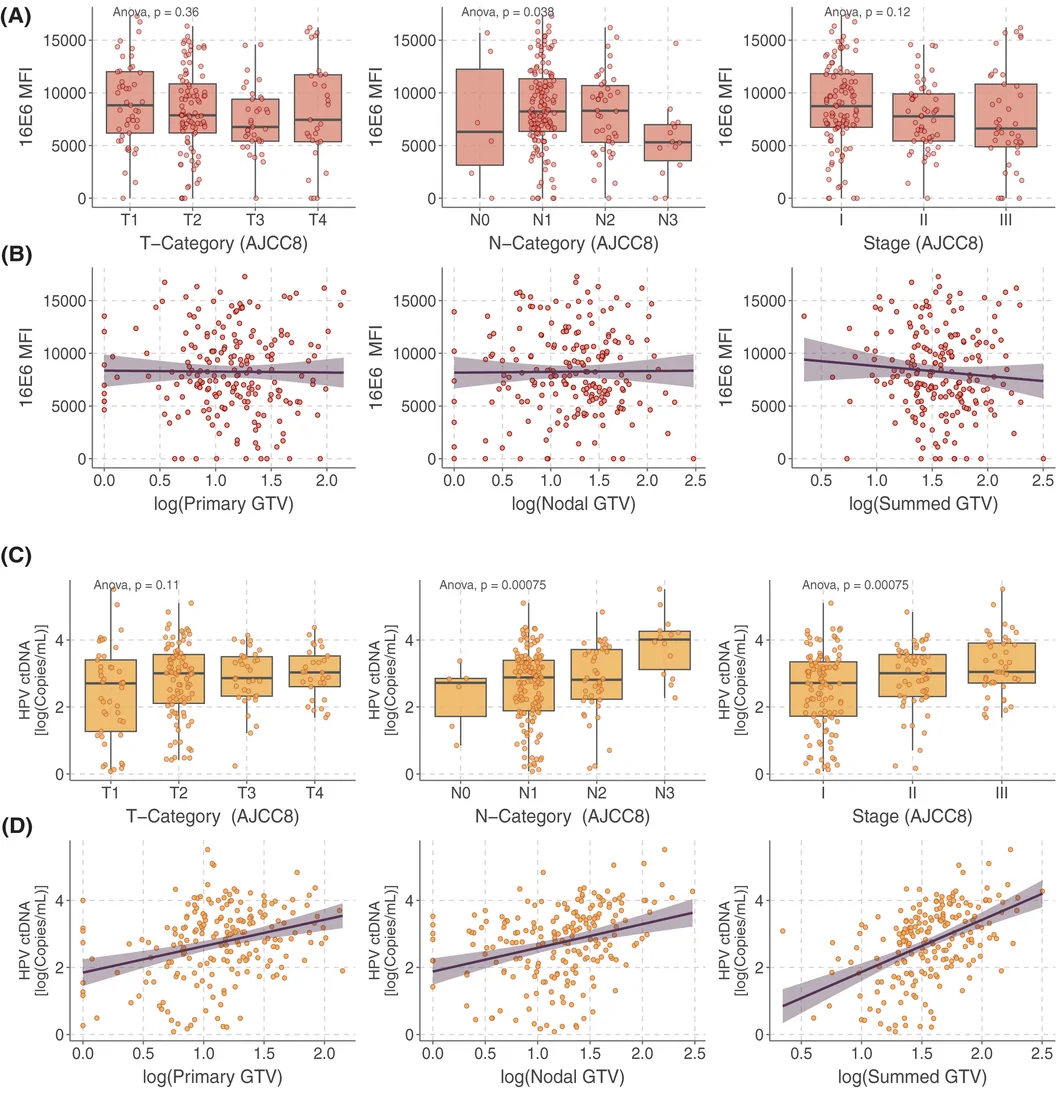
HPV ctDNA but not serology is associated with disease burden. (A) HPV16 E6 serology median fluorescence intensity (MFI) decreased significantly with increasing N‐category (ANOVA, *p* = 0.04), however there was no significant difference between HPV16 MFI and T‐category or overall stage (ANOVA, *p* > 0.1). (B) HPV16 E6 MFI demonstrated no significant association with primary, nodal, or combined GTV (*p* ≥ 0.25). (C) HPV16 ctDNA concentration significantly increases with N‐category (ANOVA, *p* = 7e‐04) and overall stage (ANOVA, *p* = 0.00068) but not T‐category (ANOVA, *p* = 0.1). (D) HPV16 ctDNA concentration demonstrated a significant positive correlation with primary GTV (Pearson, *r* = 0.30, *p* = 1.2e‐5), nodal GTV (Pearson, *r* = 0.32, *p* = 3.5e‐06), and combined GTV (Pearson, *r* = 0.48, *p* = 6.0e‐12).

Prior to Pearson correlation analysis, all samples were assessed to confirm that variables followed normal distributions and were independent of one another. Both HPV ctDNA concentration and GTV were log‐transformed to meet assumptions of normality. For visualization purposes in log‐scale plots (Figures 2 and 4), zero ctDNA values were handled by adding a small constant (0.01) prior to log‐transformation to enable plotting while preserving the logarithmic relationship for non‐zero values. Statistical tests for paired data were performed using ggpubr (v0.6.0) and were two‐sided unless otherwise stated.

Heatmaps were generated using ComplexHeatmap (v2.8.0), tables were generated using gtsummary (v1.7.2) and gt (v0.9.0), and any other general plotting functions that were used include ggplot2 (v3.4.2), ggExtra (v0.10.1), ggthemr (v1.1.0), gridExtra (v2.3), cowplot (v1.1.1).

## 3 Results

### 3.1 Overview of the OPC Cohort

We conducted a retrospective analysis of prospectively collected blood samples from 262 consecutive patients with newly diagnosed non‐metastatic p16+ OPC (Table 1). Most patients were male (84%) and the median age was 62 years at diagnosis. The majority of patients were in early stages (53% in Stage I), and a significant portion were current (21%) or ex‐smokers (43%).

**TABLE 1:** Oropharyngeal cancer patient cohort characteristics. Baseline clinical characteristics of HPV+ OPC cohort.

Characteristic	*N* = 262^a^
Age at diagnosis	62 (56, 68)
Sex	
Male	221 (84%)
Female	41 (16%)
p16 status	
Positive	262 (100%)
Overall stage (AJCC8)	
I	139 (53%)
II	67 (26%)
III	56 (21%)
T category	
T1	61 (23%)
T2	115 (44%)
T3	45 (17%)
T4	41 (16%)
N category	
N0	13 (5.0%)
N1	178 (68%)
N2	54 (21%)
N3	17 (6.5%)
Smoking status	
Non‐smoker	96 (37%)
Ex‐smoker	112 (43%)
Current	54 (21%)

^a^ Median (Q1, Q3); *n* (%).

Samples were collected pre‐treatment, and HPV antibodies and ctDNA were quantified for each patient. 242 of the 262 samples had data for both serology and HPV‐seq (Figure 2A) and formed the cohort for downstream analyses. HPV ctDNA was profiled using HPV‐seq, a hybrid capture sequencing approach that captures the whole viral genome of five high‐risk HPV genotypes (HPV16, 18, 31, 33, 35) and the E6 and E7 genes of 33 additional HPV genotypes (Table S1). HPV antibody levels were measured using a multiplex serology panel targeting antibodies for 10 HPV genotypes [16] (Table S1).

### 3.2 Detectability of HPV ctDNA and Serology

HPV ctDNA positivity and seropositivity were found in 235/242 (97%) and 216/242 (89%) of cases, respectively, according to previously described algorithms (Methods). While serology and HPV‐seq identified the same four genotypes, HPV‐seq found two additional genotypes not included in the serology panel; despite this, both assays identified HPV16 as the most prevalent genotype (203/242 ctDNA; 202/242 serology) (Figure 2B). We validated HPV‐seq results by digital PCR (ddPCR) for HPV16, HPV18, HPV33, and HPV35; the genotype result from HPV‐seq was successfully detected using ddPCR in 238/242 (98%) of patients, while the remaining four samples had insufficient material. Quantification of HPV ctDNA levels was highly correlated using the two assays (Figure 2C) (*R* ^2^ = 0.973). Ten patients with atypical presentations underwent HPV in situ hybridization to confirm HPV‐driven etiology. Nine were confirmed HPV‐positive, consistent with our HPV‐seq results (Table S2). This is consistent with the historically low p16+/HPV− discordance rate at our institution (1.5%) [15].

To evaluate the specificity of HPV ctDNA detection, we analyzed plasma from 50 healthy controls. Forty‐four (88%) had no detectable HPV ctDNA and six (12%) showed low but detectable levels, four of which were HPV16‐positive (Figure S2A). Receiver operating characteristic curve analysis demonstrated excellent discrimination between cancer patients and healthy controls (AUC = 0.975), with 94.6% sensitivity and 96% specificity at the optimal threshold of 0.61 copies/mL, as determined by Youden's index (Figure S2B,C). For this study, we prioritized sensitivity over specificity to capture the full spectrum of disease burden to evaluate ctDNA as a marker of tumor burden; therefore, we opted to include all HPV detectable samples. Optimization of specificity thresholds for population screening will require analysis of larger cohorts reflective of the general population.

### 3.3 Comparison of Genotyping Results Between HPV ctDNA and Serology

We next compared serology and ctDNA results across genotypes within individual patients. We found that patients generally had complementary detectability between ctDNA and serology (Figure 2D). In 38 cases where multiple genotypes were detected in either serology or HPV‐seq, we plotted the proportion of each patient's dominant genotype relative to their total signal (Figure 3A). While serology demonstrated variable dominant signal, all but two patients had HPV ctDNA reads aligned to at least 99.4% of the dominant genotype.

In many of the serology cases that contained multiple genotypes, HPV16 and HPV33 were detected together (Figure 3B). Certain HPV antibodies are known to be cross‐reactive within the multiplex serology panel. To identify biomarkers that were the least cross‐reactive, we performed a correlation analysis on the E6, E7, and L1 antibodies across high‐risk HPV genotypes (HPV16, HPV18, HPV31, HPV33, HPV35). Antibodies for HPV16 L1 and E7 were the most cross‐reactive, with a significant positive correlation between genotypes (Figure 3C). Interestingly, serology for HPV16 E6 demonstrated little to no cross‐reactivity between other HPV genotypes. The weaker E6 cross‐reactivity observed among genotypes may reflect the limited sample size for these rarer genotype subgroups, warranting further investigation in larger cohorts.

### 3.4 Sensitivity of HPV16 Serology

Because HPV16 is the most prevalent genotype in OPC, we next compared various HPV16 antibodies (E1, E2, E4, E6, E7, and L1) and HPV16 ctDNA in our cohort (Figure 3D). No significant correlations were observed between the abundance of these antibodies and ctDNA concentration (*p* ≥ 0.3 for all). Among the 242 patients with detectable HPV16 ctDNA (*n* = 203), HPV16 E6 seropositivity demonstrated the highest sensitivity (96%), while E1 (67%), E4 (33%), E7 (72%), and L1 (48%) demonstrated considerably lower sensitivities (Figure 3E and Figure S1). These findings reflect that HPV16 E6 antibody levels are best suited to compare against HPV16 ctDNA.

### 3.5 HPV ctDNA but not HPV Serology Is Positively Correlated With Disease Burden

We sought to explore the association between HPV16 E6 antibody levels and HPV ctDNA concentration as it related to disease burden. Disease burden was assessed using standard TNM‐8 classification as well as using volumetric assessment of the gross tumor volume (GTV) from both the primary tumor and any involved lymph nodes. HPV16 E6 antibody levels were significantly lower with advancing N‐category (*p* = 0.04, ANOVA); however, there were no associations with GTV (Pearson, *p* ≥ 0.25 for all) (Figure 4A,B). In contrast, HPV16 ctDNA demonstrated a significant positive association with both N‐category (*p* = 7e‐04) and overall stage (*p* = 7e‐4), was positively correlated with both primary and nodal GTV, but demonstrated the best correlation with combined GTV (*r* = 0.48, *p* = 6.0e‐12) (Figure 4C,D).

We suspected that the weak inverse association between HPV16 E6 antibody levels and disease burden may be explained by immunosuppression that can develop in patients with advanced cancer. Pre‐treatment complete blood counts were available for 241/242 (99.6%) patients in the cohort. Lymphocyte counts were negatively associated with T‐category (*p* = 0.047), overall stage (*p* = 0.028), primary GTV (Pearson, r = −0.13, *p* = 0.048), and HPV ctDNA abundance (Pearson, r = −0.14, *p* = 0.027), but there was no statistically significant association with HPV16 E6 antibody levels (Figure S3). Additionally, the neutrophil‐to‐lymphocyte ratio (NLR), which has previously been used as a marker of poor immunologic health in cancer patients [18], was positively correlated with overall stage (*p* = 0.0022) and had a positive trend with T‐category (*p* = 0.064) (Figure S3).

Finally, we evaluated whether HPV ctDNA and serology provided independent predictive value for disease burden (i.e., summed GTV). In a linear model incorporating both HPV ctDNA concentration and HPV16 E6 antibody levels, only HPV ctDNA was a significant covariate. These findings highlight that HPV ctDNA is potentially more reflective of OPC disease burden and has the potential to better risk‐stratify p16+ OPC patients compared to HPV serology.

## 4 Discussion

Biomarkers for HPV+ OPC have long been sought for early interception and risk stratification. Here, we describe the largest and most comprehensive comparison of HPV serology and HPV ctDNA in a cohort of p16+ OPC patients. While both assays could detect dominant oncogenic HPV genotypes, HPV‐seq proved more sensitive. HPV serology but not HPV ctDNA demonstrated a high degree of cross‐reactivity between HPV genotypes. In contrast to E6 serology, HPV ctDNA was associated with higher disease burden, agreeing with previous findings [10, 19]. Cross‐reactivity of certain antibodies hinders the genotype specificity of HPV serology, while HPV16 E6 demonstrates relatively low cross‐reactivity with other HPV genotypes. Here, 97% of p16+ patients had detectable HPV ctDNA, and levels were significantly associated with N‐category, overall stage, and tumor volume. We also found that the overwhelming majority of cases detected by HPV‐seq had 100% of sequenced fragments aligned to one genotype. Consistent with our results, multiple genotypes are rarely detected in individual OPC cases [20, 21, 22] unlike in cervix cancer where co‐infection of multiple HPV genotypes is more common [23]. Given the prognostic role of HPV genotype in OPC [24], direct interrogation of HPV genotype from peripheral blood may have added utility.

Immunosurveillance plays a critical role in the pathogenesis of HPV+ OPC. We found that host immunity—through absolute lymphocyte count and NLR—was weakly associated with high disease burden and HPV ctDNA levels but not with HPV16 E6 antibody levels. Interestingly, HPV16 E6 antibody levels were lower in certain patients with more advanced locoregional disease in whom HPV ctDNA levels were readily detectable. Notably, the discordance between serology and ctDNA—where antibodies often detect multiple HPV genotypes while ctDNA is genotype‐specific—reflects their distinct biological origins: antibodies represent cumulative immunological memory from past and present HPV exposures, whereas ctDNA reflects real‐time shedding exclusively from active tumor cells. Future studies could characterize distinct mechanisms by which lymphocytes influence HPV serologic response during OPC tumorigenesis [25, 26, 27] deciphering the relationship between persistent HPV infection, immune‐mediated clearance, and the production of HPV antibodies and ctDNA.

To date, there remain no established screening strategies for HPV+ OPC despite the rapid increase in incidence. HPV serology is a cost‐effective and scalable assay that may be effective as a population screening tool. HPV16 E6 seropositivity is associated with greater than 200‐fold increased risk of developing OPC [28] and an excellent negative predictive value [9]. However, long lead times to diagnosis and low positive predictive value indicate that additional investigations would be needed.

HPV ctDNA may be complementary to serology by reporting HPV genotype, decreasing lead time to diagnosis, and reflecting disease burden. Recent publications report improved diagnostic accuracy of ctDNA needed to screen compared to traditional serology or ddPCR‐based assays [29]. Additionally, Das et al. [30] demonstrated that circulating tumor HPV DNA can be detected up to 10.3 years prior to OPC diagnosis, substantially extending the window for early interception. These findings support the integration of ctDNA and serological biomarkers as a feasible approach for preclinical screening, potentially enabling detection of asymptomatic, early‐stage HPV+ OPC.

We propose a two‐tier screening strategy for future investigation that leverages the complementary strengths of HPV serology and ctDNA detection to optimize both clinical performance and cost‐effectiveness. HPV serology represents a well‐documented, highly scalable assay that has been validated across multiple large studies and is suitable for population‐level screening. Serological assays for HPV antibodies are high‐throughput, automatable, and relatively inexpensive (approximately $35USD per sample), with the potential to be cheaper with a smaller panel size. In a proof‐of‐concept study utilizing HPV16 E6 serology for early detection of OPC in asymptomatic individuals, Busch et al. reported ≥ 90% sensitivity and 99% specificity with negative predictive value in the general population exceeding 99.9%, though the positive predictive value was modest at approximately 10%–20% [5]. In contrast, HPV ctDNA detection through HPV‐seq provides definitive viral genotyping and dynamic quantification of tumor burden, but at higher cost (approximately $145USD per sample) due to next‐generation sequencing requirements. By using serology for initial population‐level screening and restricting the more expensive ctDNA testing to individuals who screen antibody‐positive over a defined period, this approach could substantially reduce per‐capita screening costs while maintaining high sensitivity. This tiered strategy mirrors successful implementations in other cancer screening programs, such as the use of fecal immunochemical testing followed by colonoscopy for colorectal cancer screening, which has demonstrated a 33% reduction in colorectal cancer mortality in a cohort of over 2 million individuals [31]. Furthermore, the genotype‐specific nature of ctDNA detection provides critical information for clinical management that cannot be obtained from serology alone, potentially justifying the additional cost for confirmatory testing in screen‐positive individuals.

Our findings demonstrating the complementary roles for HPV serology and ctDNA detection in OPC are consistent with the broader literature across HPV‐associated malignancies. HPV antibody serology has shown prognostic and screening utility in anogenital cancers: in a prospective cohort, pre‐diagnostic HPV16 E6 seropositivity was significantly associated with future risk of anal cancers, establishing serology as a potential early biomarker of HPV‐driven anal carcinogenesis [32]. Furthermore, HPV ctDNA levels in anal cancer correlate with disease burden, decrease with treatment response, and predict recurrence across both definitive and palliative settings via ddPCR [33, 34] and NGS‐based approaches [35, 36]. In cervical cancer, pre‐diagnostic HPV16 E6 seropositivity is a strong predictor of invasive disease, yet low seroprevalence of 11% among cervical cancer cases limits its standalone utility as a screening biomarker [37]. HPV ctDNA could offer complementary sensitivity, as it is detectable at baseline in ≥ 95% of patients with both early‐ and late‐stage disease [10, 38], and persistent HPV ctDNA following chemoradiation independently predicted inferior progression‐free survival with a median lead time of 5.9 months [10]. Collectively, these findings suggest that a strategy combining antibody‐based screening with confirmatory HPV ctDNA testing may have broader applicability beyond OPC.

Our study has limitations that warrant consideration. First, only a subset of samples had tumor HPV nucleic acid testing conducted. Additionally, the relatively small number of healthy controls limits our ability to establish validated thresholds that would apply for population screening. Furthermore, our study cohort consisted predominantly of white North American males, which may not fully represent the broader population affected by this disease. Future validation studies incorporating a more diverse cohort of head and neck cancer patients will be important to accurately assess the generalizability of our findings.

Taken together, our findings have potential implications for early detection and prognostication of HPV+ OPC and highlight opportunities for future studies incorporating both serological and ctDNA biomarkers.

## Author Contributions

**Lucas Penny:** conceptualization, writing – original draft, writing – review and editing, visualization, methodology, formal analysis. **Eric Yang Stutheit‐Zhao:** methodology, writing – review and editing. **Birgitta E. Michels:** methodology, data curation. **Fabian Rosing:** methodology, data curation, writing – review and editing. **Zhen Zhao:** methodology, data curation, writing – review and editing. **Yangqiao Zheng:** writing – review and editing, methodology, data curation. **Jinfeng Zou:** writing – review and editing, methodology, software. **Shao Hui Huang:** writing – review and editing, data curation. **Johnny Carlton:** writing – review and editing, data curation. **Andrew McPartlin:** writing – review and editing, data curation. **John R. de Almeida:** data curation. **David Goldstein:** writing – review and editing, data curation. **Andrew Hope:** writing – review and editing, data curation. **Ali Hosni:** writing – review and editing, data curation. **John Kim:** writing – review and editing, data curation. **Fei‐Fei Liu:** writing – review and editing, data curation. **C. Jillian Tsai:** writing – review and editing, data curation. **John N. Waldron:** data curation. **Anna Spreafico:** writing – review and editing, data curation. **Enrique Sanz‐Garcia:** writing – review and editing, data curation. **Lillian L. Siu:** data curation, writing – review and editing. **Tim Waterboer:** writing – review and editing, conceptualization, methodology, resources, project administration, investigation, funding acquisition. **Geoffrey Liu:** writing – review and editing, project administration, resources, methodology, conceptualization, investigation, funding acquisition. **Scott V. Bratman:** writing – review and editing, conceptualization, resources, supervision, investigation, funding acquisition, project administration, methodology.

## Ethics Statement

Ethics approval was obtained from the UHN ethics board (REB#07–0521) and informed consent was provided by every patient.

## Conflicts of Interest

Z.Z., J.Z., and S.V.B. are co‐inventors of a patent application pending related to detection of HPV circulating cell‐free DNA. It is held by the institution and is not royalty generating. Unrelated to this work, S.V.B. is inventor on patents related to cell‐free DNA mutation and methylation analysis technologies that have been licensed to Roche Molecular Diagnostics and Adela, respectively. S.V.B. is a co‐founder of, has ownership in, and serves in a leadership role at Adela. A.H. holds a non‐financial leadership role in the Liver TSG at the Elekta MRL Consortium and has received an investigator‐initiated grant from Elekta for partial funding of the ULTRAS Phase III RCT. C.J.T. has served as a consultant for Varian Medical Inc. since 2020. A.S. has served as a compensated consultant or advisory board member for Merck, Bristol‐Myers Squibb, GSK, BeiGene, and Pfizer, and as a non‐compensated advisory board member for Alentis and Boehringer Ingelheim. A.S. has received grant or research support from clinical trials sponsored by Novartis, Bristol‐Myers Squibb, Symphogen, AstraZeneca/Medimmune, Merck, Bayer, Surface Oncology, Janssen Oncology/Johnson and Johnson, Roche, Regeneron, Alkermes, Array Biopharma/Pfizer, GSK, NuBiyota, Oncorus, Treadwell, Amgen, ALX Oncology, Genentech, Seagen, Servier, Incyte, Alentis, Teva Therapeutics, Merus, EMD Serono, and Auricula Biosciences. L.L.S. has received research grants from Bristol‐Myers Squibb, Roche/Genentech, GSK, Merck, Novartis, Pfizer, AstraZeneca, Boehringer‐Ingelheim, Bayer, Amgen, Abbvie, EMD Serono, Daiichi Sankyo, Gilead, Marengo, Incyte, Legochem, and Loxo/Lilly, and has received honoraria or consultation fees from Merck, Roche, Voronoi, GSK, Bayer, Pfizer, Arvinas, Daiichi Sankyo, Amgen, Pangea, Tubulis, LIZ Therapeutics, Marengo, AstraZeneca, Incyte, Gilead, Bristol‐Myers Squibb, Systimmune, and EMD Serono. L.L.S. partner is a co‐founder of Treadwell Therapeutics. J.R.A. has received grant funding from Cardinal Health. E.S.‐G. has received consulting fees from GSK and Incyte, and has received research grants from RGT and GSK for clinical trials. T.W. serves on an uncompensated advisory board for Merck, Sharp & Dohme (MSD), with this relationship continuing. All other authors have no conflicts of interest to declare.

## Supporting information


**Figure S1:** Cross Reactivity and Sensitivity of Serology. (A) The heatmap illustrates cross‐reactivity among HPV genotypes for the E6, E7, and L1 oncoproteins, where the size and color intensity of each circle represent the strength of Pearson correlation coefficients between serological responses. (B) Boxplots display the distribution of Pearson correlations (cross‐reactivity) for E6, E7, and L1 across various HPV genotypes. (C) Scatter plots depict the relationship between HPV16 E6 and E7 mean fluorescence intensity (MFI) values and circulating tumor DNA (ctDNA) concentrations.
**Figure S2:** Comparison of HPV ctDNA levels in oropharyngeal cancer patients and healthy controls. (A) Distribution of HPV ctDNA levels (log10 Seq Copies/mL) in oropharyngeal cancer patients and healthy controls. Box plots show median, interquartile range, and 1.5 × interquartile range. (B) Discriminative performance of HPV ctDNA detection between cancer patients and healthy controls (AUC = 0.975). (C) Histogram of HPV ctDNA levels across cancer patients and healthy controls. Dashed line: optimal cut point.
**Figure S3:** Peripheral blood counts association with disease burden, HPV ctDNA, and serology. (A) Distribution of absolute lymphocyte levels and NLRs across different tumor (T) categories, nodal (N) stages, and overall disease stage. (B) Correlation between various peripheral blood counts, including lymphocytes, neutrophils, hemoglobin (Hb), monocytes, and platelets, and key disease metrics such as HPV16 E6, ctDNA concentration, and gross tumor volume (GTV).

**Table S1:** Description of all genotypes detected with HPV‐seq and serology.
**Table S2:** Institutional validation of HPV status in 10 patients with atypical presentations.
**Table S3:** Sequencing coverage and quality statistics. Sequencing files were aligned to reference genome Hg19.
**Table S4:** Serology cutoff values for HPV antibody positivity.

## Data Availability

All source code is publicly available on GitHub (https://github.com/lucasjpenny/serology_manuscript). Further data that support the findings of this study are available from the corresponding author upon reasonable request.
