# Supplementary material for: HPV Serology and Circulating Viral DNA for Detection, Genotyping, and Measurement of Disease Burden in Oropharyngeal Cancer
Source: Int J Cancer. 2026 Jun 30;159(9):2336–46. doi: 10.1002/ijc.70619 (PMC13547982; doi:10.1002/ijc.70619)
Supplement: Supplementary file 1 [file IJC-159-2336-s001.pdf]

Title

HPV Serology and Circulating Viral DNA for Detection, Genotyping, and Measurement of Disease Burden in Oropharyngeal Cancer

Lucas Penny, Eric Yang Stutheit-Zhao, Birgitta E. Michels, Fabian Rosing, Zhen Zhao, Yangqiao Zheng, Jinfeng Zou, Shao Hui Huang, Johnny Carlton, Andrew McPartlin, John R. de Almeida, David Goldstein, Andrew Hope, Ali Hosni, John Kim, Fei-Fei Liu, C Jillian Tsai, John N. Waldron, Anna Spreafico, Enrique Sanz Garcia, Lillian L. Siu, Tim Waterboer, Geoffrey Liu, Scott V. Bratman

Table of Contents

Figure S1: Cross Reactivity and Sensitivity of Serology.

Figure S2: Comparison of HPV ctDNA levels in oropharyngeal cancer patients and healthy controls.

Figure S3: Peripheral blood counts association with disease burden, HPV ctDNA, and serology.

Supplementary Tables S1 – S4: available in the separate excel file.

Figure S1: Cross Reactivity and Sensitivity of Serology.

(A) The heatmap illustrates cross-reactivity among HPV genotypes for the E6, E7, and L1 oncoproteins, where the size and color intensity of each circle represent the strength of Pearson correlation coefficients between serological responses.

(B) Boxplots display the distribution of Pearson correlations (cross-reactivity) for E6, E7, and L1 across various HPV genotypes.

(C) Scatter plots depict the relationship between HPV16 E6 and E7 mean fluorescence intensity (MFI) values and circulating tumor DNA (ctDNA) concentrations.

Figure S2: Comparison of HPV ctDNA levels in oropharyngeal cancer patients and healthy controls.

(A) Distribution of HPV ctDNA levels (log10 Seq Copies/mL) in oropharyngeal cancer patients and healthy controls. Box plots show median, interquartile range, and 1.5× interquartile range.

(B) Discriminative performance of HPV ctDNA detection between cancer patients and healthy controls (AUC=0.975).

(C) Histogram of HPV ctDNA levels across cancer patients and healthy controls. Dashed line: optimal cut point.

Figure S3: Peripheral blood counts association with disease burden, HPV ctDNA, and serology.

(A) Distribution of absolute lymphocyte levels and NLRs across different tumor (T) categories, nodal (N) stages, and overall disease stage.

(B) Correlation between various peripheral blood counts, including lymphocytes, neutrophils, hemoglobin (Hb), monocytes, and platelets, and key disease metrics such as HPV16 E6, ctDNA concentration, and gross tumor volume (GTV)

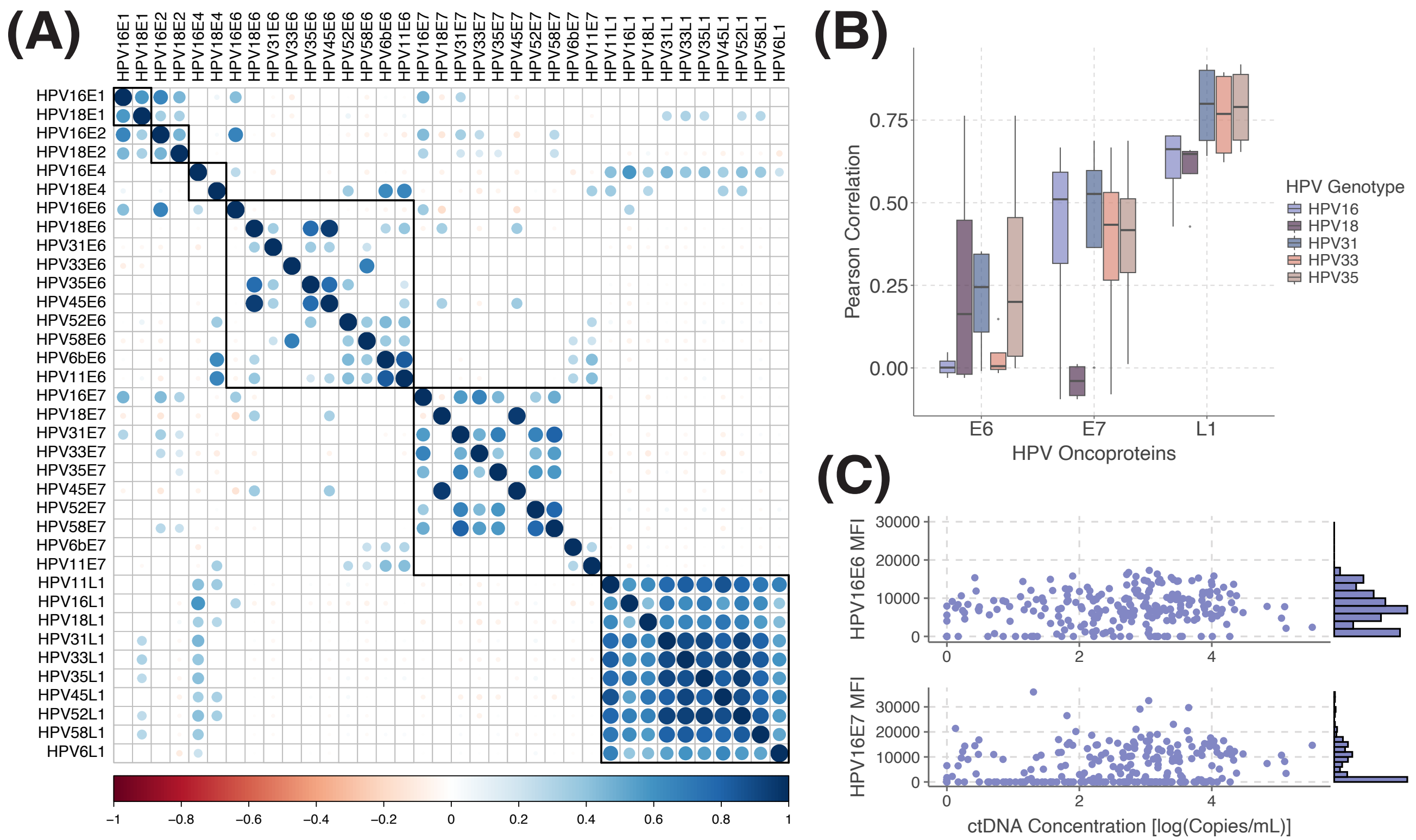

(A)
HPV16E1
HPV18E1
HPV16E2
HPV18E2
HPV16E4
HPV18E4
HPV16E6
HPV18E6
HPV31E6
HPV33E6
HPV35E6
HPV45E6
HPV52E6
HPV58E6
HPV6bE6
HPV11E6
HPV16E7
HPV18E7
HPV31E7
HPV33E7
HPV35E7
HPV45E7
HPV52E7
HPV58E7
HPV6bE7
HPV11E7
HPV11L1
HPV16L1
HPV18L1
HPV31L1
HPV33L1
HPV35L1
HPV45L1
HPV52L1
HPV58L1
HPV6L1
−1
−0.8
−0.6
−0.4
−0.2
0
0.2
0.4
0.6
0.8
1
(B)
Pearson Correlation
0.00
0.25
0.50
0.75
E6
E7
L1
HPV Oncoproteins
HPV Genotype
HPV16
HPV18
HPV31
HPV33
HPV35
(C)
HPV16E6 MFI
30000
20000
10000
0
HPV16E7 MFI
30000
20000
10000
0
0
2
4
ctDNA Concentration [log(Copies/mL)]

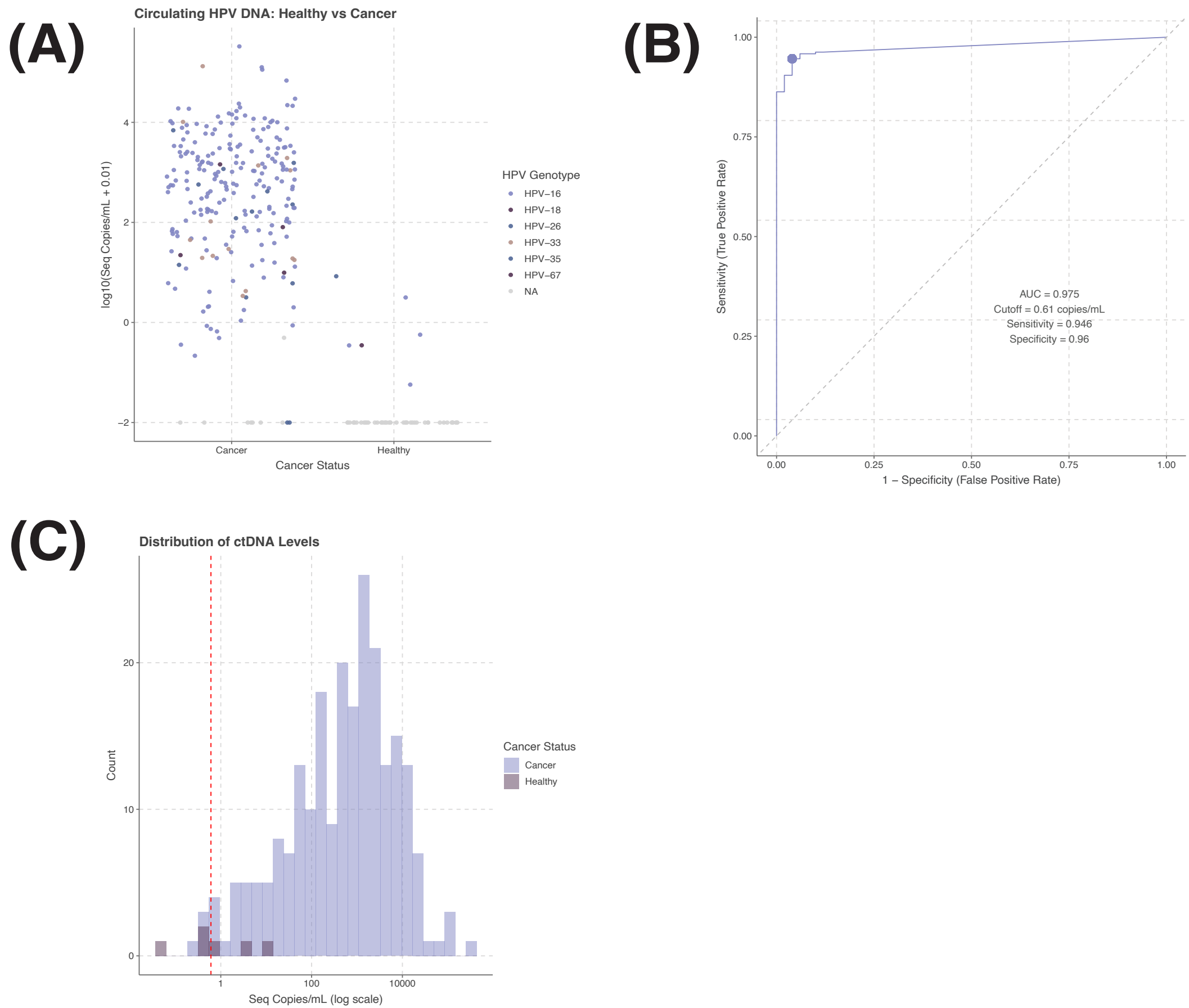

(A)
Circulating HPV DNA: Healthy vs Cancer
log10(Seq Copies/mL + 0.01)
4
2
0
−2
Cancer
Healthy
Cancer Status
HPV Genotype
HPV−16
HPV−18
HPV−26
HPV−33
HPV−35
HPV−67
NA
(B)
Sensitivity (True Positive Rate)
1.00
0.75
0.50
0.25
0.00
0.00
0.25
0.50
0.75
1.00
1 − Specificity (False Positive Rate)
AUC = 0.975
Cutoff = 0.61 copies/mL
Sensitivity = 0.946
Specificity = 0.96
(C)
Distribution of ctDNA Levels
Count
20
10
0
1
100
10000
Seq Copies/mL (log scale)
Cancer Status
Cancer
Healthy

(A)

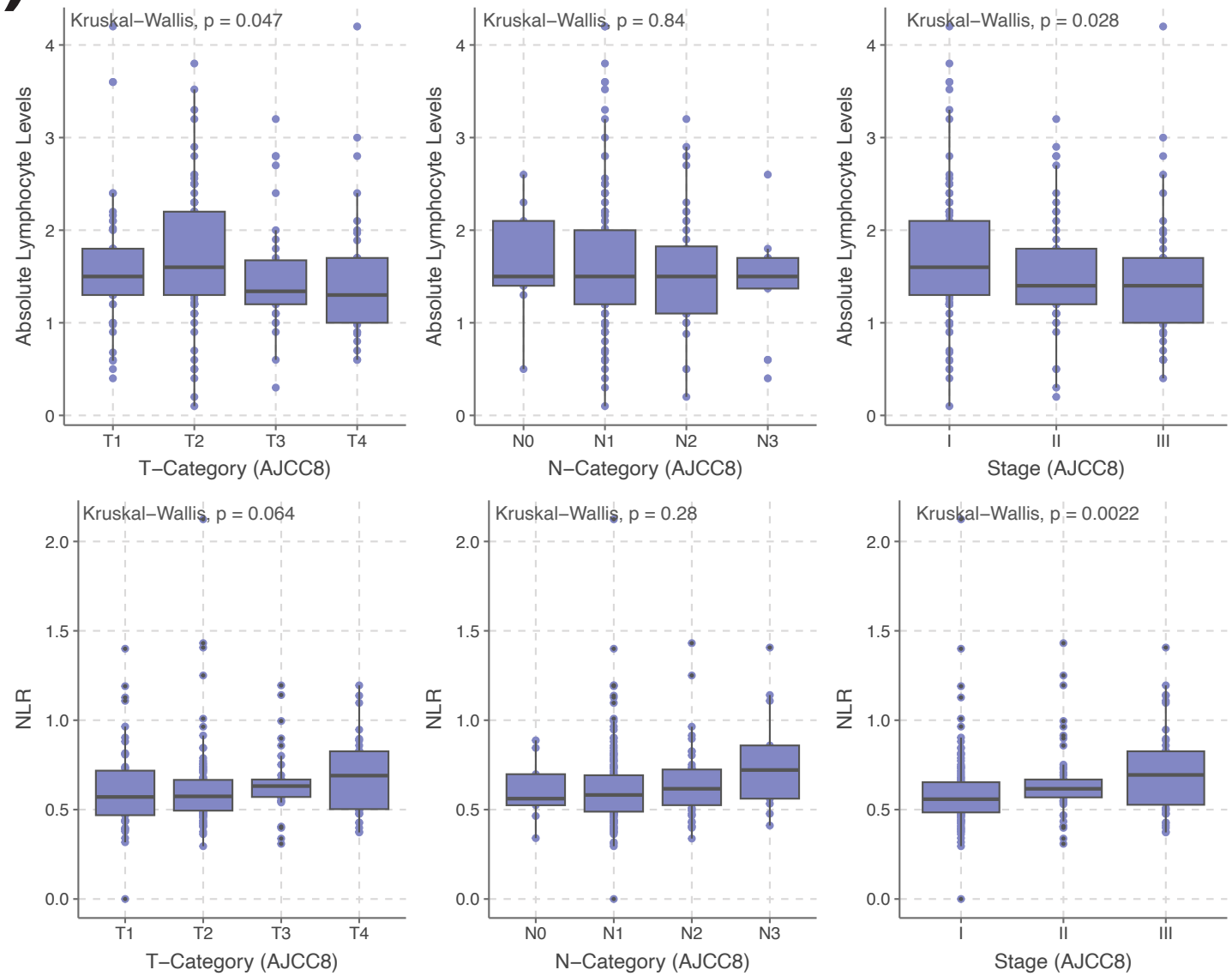

Kruskal–Wallis, p = 0.047
Kruskal–Wallis, p = 0.84
Kruskal–Wallis, p = 0.028
Absolute Lymphocyte Levels
T1
T2
T3
T4
T–Category (AJCC8)
N0
N1
N2
N3
N–Category (AJCC8)
I
II
III
Stage (AJCC8)
Kruskal–Wallis, p = 0.064
Kruskal–Wallis, p = 0.28
Kruskal–Wallis, p = 0.0022
NLR
T–Category (AJCC8)
N–Category (AJCC8)
Stage (AJCC8)

(B)

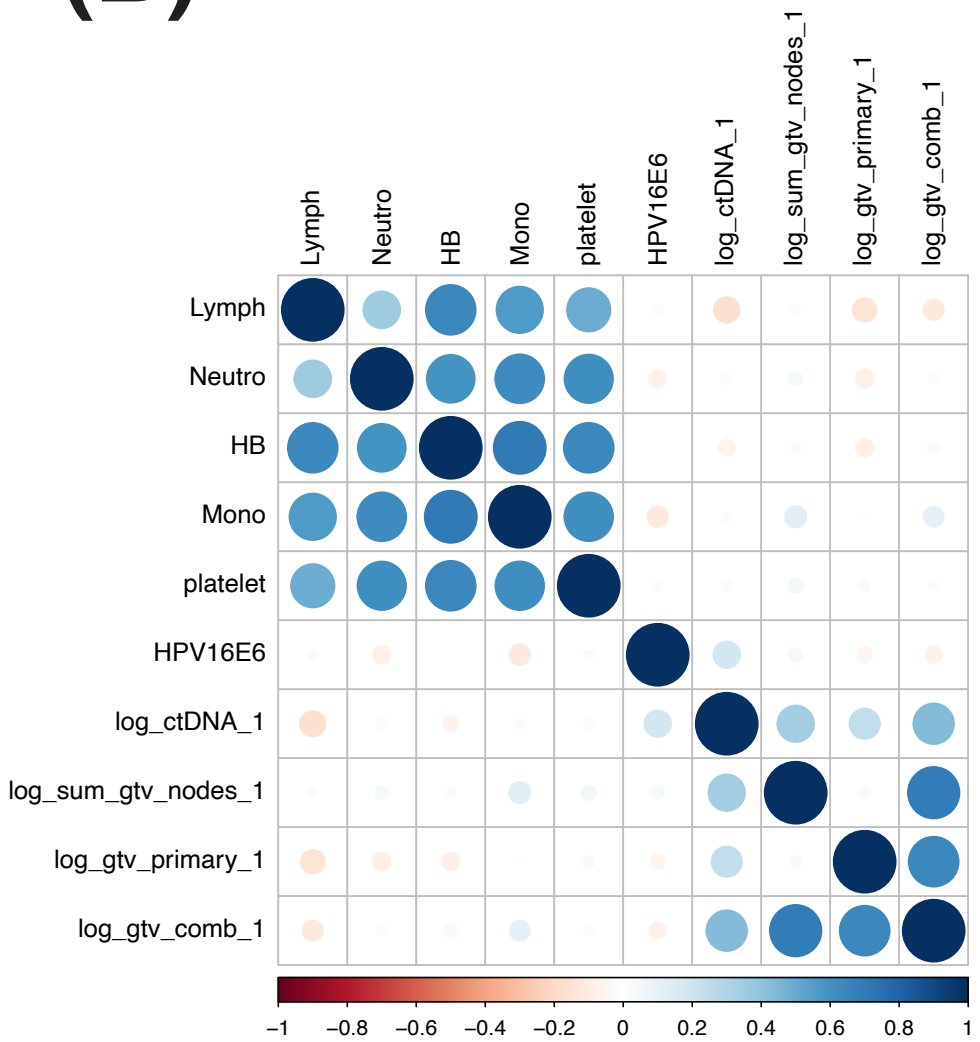

Lymph
Neutro
HB
Mono
platelet
HPV16E6
log_ctDNA_1
log_sum_gtv_nodes_1
log_gtv_primary_1
log_gtv_comb_1
−1
−0.8
−0.6
−0.4
−0.2
0
0.2
0.4
0.6
0.8
1
